# Percutaneous hepatic melphalan perfusion: Single center experience of procedural characteristics, hemodynamic response, complications, and postoperative recovery

**DOI:** 10.1371/journal.pone.0254817

**Published:** 2021-07-16

**Authors:** Manuel Florian Struck, Peter Kliem, Sebastian Ebel, Alice Bauer, Holger Gössmann, Rhea Veelken, Florian van Bömmel, Timm Dennecke, Sebastian N. Stehr, Felix F. Girrbach

**Affiliations:** 1 Department of Anesthesiology and Intensive Care Medicine, University Hospital Leipzig, Leipzig, Saxony, Germany; 2 Department of Diagnostic and Interventional Radiology, University Hospital Leipzig, Leipzig, Saxony, Germany; 3 Division of Hepatology, Department of Medicine II, University Hospital Leipzig, Leipzig, Saxony, Germany; Ohio State University Wexner Medical Center Department of Surgery, UNITED STATES

## Abstract

**Background:**

Percutaneous hepatic melphalan perfusion (PHMP) for the selective treatment of hepatic metastases is known to be associated with procedural hypotension and coagulation disorders. Studies on anesthetic management, perioperative course, complications, and postoperative recovery in the intensive care unit (ICU) have not been published.

**Methods:**

In a retrospective observational study, we analyzed consecutive patients who were admitted for PHMP over a 6-year period (2016–2021). Analyses included demographic, treatment, and outcome data with regard to short-term complications until ICU discharge.

**Results:**

Fifty-three PHMP procedures of 16 patients were analyzed. In all of the cases, procedure-related hypotension required the median (range) highest noradrenaline infusion rate of 0.5 (0.17–2.1) μg kg min^-1^ and fluid resuscitation volume of 5 (3–14) liters. Eighty-four PHMP-related complications were observed in 33 cases (62%), of which 9 cases (27%) involved grade III and IV complications. Complications included airway constriction (requiring difficult airway management), vascular catheterization issues (which resulted in the premature termination of PHMP, as well as to the postponement of PHMP and to the performance of endovascular bleeding control after PHMP), and renal failure that required hemodialysis. Discharge from the ICU was possible after one day in most cases (n = 45; 85%); however, in 12 cases (23%), prolonged mechanical ventilation was required. There were no procedure-related fatalities.

**Conclusions:**

PHMP is frequently associated with challenging cardiovascular conditions and complications that require profound anesthetic skills. For safety reasons, PHMP should only be performed in specialized centers that provide high-level hospital infrastructures and interdisciplinary expertise.

## Background

Percutaneous hepatic melphalan perfusion (PHMP) is a third-line treatment option in patients with unresectable, isolated liver metastases or primary liver tumors [1–16]. Liver metastases and liver cancer tissues are primarily supplied by branches of the hepatic artery, whereas normal hepatocytes are supplied by the portal vein. In this minimally invasive procedure, high-dose melphalan (3 mg kg^-1^ ideal body weight) is directly injected into the hepatic artery via an intraarterial catheter under the use of general anesthesia. To minimize systemic toxic effects, venous blood is drained through a double-balloon catheter in the inferior cava vein and melphalan is removed by using a specific extracorporeal hemofiltration circuit. The purified blood is then returned to the systemic circulation via a second central venous catheter (CVC) that is placed in the internal jugular vein, which allows for a higher dosing of melphalan to increase chemotherapeutic effectiveness. The wash-out of melphalan at the end of the procedure is achieved by the continuation of extracorporeal filtration for 30 minutes after the end of the last melphalan infusion. The procedure can be performed as a single intervention or can be repeated, depending on the clinical condition of the patient and the patient’s response to the therapy.

Although recent studies have shown promising long-term results, PHMP itself is reported to be associated with severe cardiovascular issues and potentially life-threatening adverse events, including myocardial infarction, cerebral stroke, and bleeding complications [1, 3–15]. Considerable hemodynamic instability can frequently occur after engagement of the filters and the clamping of the bypass circuit [3, 13]. This phenomenon is largely attributed to the filtration of catecholamines, but inadvertent systemic melphalan effects have also been discussed [17]. Due to tight anticoagulation for clotting prevention, bleeding complications at the puncture sites are also possible. The filtration of cellular blood components by chemofilters can typically lead to anemia and thrombopenia in the immediate postoperative period, whereas leukopenia due to melphalan-induced bone marrow suppression is usually observed after the procedure [7].

Studies on anesthetic management and vital functions during PHMP and consecutive intensive care unit (ICU) stays are not currently available. Furthermore, the prevalence and consequences of iatrogenic complications related to vascular access, melphalan administration, and anticoagulation treatment have not been systematically explored. Thus, in the present study, we analyzed anesthetic management, procedural characteristics, complications, and the postoperative recovery of PHMP patients until ICU discharge.

## Methods

### Setting

After approval of the Ethics Committee at the Medical Faculty, Leipzig University, Leipzig, Germany (IRB00001750, project ID 500/20-ek, November 12, 2020, chair: Prof. Dr. Dr. Ortrun Riha), the database of the University Hospital Leipzig was reviewed for patients who underwent PHMP with the use of the CHEMOSAT^®^ system (Delcath Systems Inc., New York, NY, USA) between 01/2016 and 3/2021. All of the data were retrospectively collected from electronic patient records and were fully anonymized before analysis. Consent to participate was waived by the Ethics Committee due to the retrospective nature of the study.

### General management

All procedures were performed in general anesthesia by consultants and senior specialists. The patients who were scheduled for PHMP underwent inductions of general anesthesia, tracheal intubation, and mechanical ventilation. Central venous catheterizations (involving the placement of 5-Lumen CVC with one high-flow lumen and a 12 F sheath) of the right internal jugular vein were performed under ultrasound guidance. For hemodynamic management, noradrenaline infusion and a rapid infusion system were provided, whereas hypotension was mainly controlled by the adjustment of noradrenaline dosage. Infusion volumes were administered at the discretion of the attending anesthetist. Arterial lines and femoral introducers were placed by the interventional radiologist. Details on the technical steps of the PHMP procedure have been previously described [1, 2]. After the completion of all of the vascular accesses (and before the initiation of the extracorporeal circulation), patients received a bolus of 25.000 units heparin (goal activated clotting time [ACT] >450 seconds). Due to the predictable decrease in arterial blood pressure that occurred immediately after engagement of the chemofilters, most anesthesiologists aimed for systolic blood pressures (SBPs) >150 mmHg prior to starting the extracorporeal circulation. Anticoagulation was monitored by the perfusionist by using repeated ACT measurements throughout the procedure, and repeated doses of heparin were given, if necessary.

In case of an uneventful procedure, the patients were extubated in the fluoroscopic suite. All of the patients were routinely monitored for at least one day in the ICU. Vascular catheters (CVCs and arterial introducers) remained in place until the normalization of coagulation was achieved.

Laboratory samples were taken prior PHMP, at the time of ICU admission, at frequent intervals during the ICU stay, and before ICU discharge. Blood products were transfused according to local ICU standards. Antiemetic and antipyretic substances were administered when required. For the prevention of tumor lysis syndrome, oral allopurinol was administered for the first three postoperative days.

### Parameter selection

Analyses of the perioperative period and ICU stay included vital parameters (e.g., SBP, heart rate, and body temperature), management (e.g., fluid volumes and noradrenaline dosages), laboratory course, procedural times, and complications (Tables 1–3).

**Table 1:** Patient’s characteristics of percutaneous hepatic melphalan perfusion.

Parameter	
Female; n (%)	11 (69)
Male; n (%)	5 (31)
Age, years; median (range)	60 (28–81)
BMI, kg/m^2^, median (range)	26 (20–37)
ASA II; n (%)	14 (88)
ASA III; n (%)	2 (12)
Underlying disease	
• Uveal melanoma; n (%)	11 (69)
• Cholangiocarcinoma; n (%)	3 (19)
• Hepatocellular carcinoma; n (%)	1 (6)
• Tonsillar carcinoma; n (%)	1 (6)
Previous treatments of liver metastases prior to PHMP	
• Surgery only; n (%)	1 (6)
• Surgery and brachytherapy; n (%)	1 (6)
• Surgery and systemic chemotherapy; n (%)	1 (6)
• SIRT and TACE; n (%)	1 (6)
• Brachytherapy only; n (%)	2 (13)
• No previous treatments; n (%)	10 (63)
Number of PHMP procedures; median (range)	2 (1–6)
Balanced anesthesia; n (%)	44 (83)
TIVA; n (%)	9 (17)
LOS at the ICU, days; median (range)	1 (1–3)
30-days mortality; n (%)	0 (0)

BMI, body mass index; ASA, American Society of Anesthesiologists classification; PHMP, percutaneous hepatic melphalan perfusion; SIRT, selective internal radiation therapy; TACE, trans-arterial chemoembolization; TIVA, total intravenous anesthesia; LOS, length of stay; ICU, intensive care unit.

**Table 2:** Procedure times of percutaneous hepatic melphalan perfusion.

Parameter	Minutes, median (range)
Anesthesia induction and CVC placement	35 (20–85)
Intervention time	160 (100–248)
Extracorporeal circulation time	110 (80–170)
Wakeup time	15 (9–24)
Total time	224 (165–332)

CVC, central venous catheterization

**Table 3:** Severe perioperative complications and adverse events until intensive care unit discharge.

Procedure	Complication	Clavien Dindo
#11	Massive facial edema/mucosal bleeding requiring prolonged mechanical ventilation and oral tamponade	Grade IIIb
#22	Cervical hematoma and neck swelling requiring difficult airway management re-ETI and angiography intervention	Grade IVa
#26	Cervical hematoma and neck swelling requiring prolonged mechanical ventilation	Grade IIIb
#30	Massive facial edema requiring prolonged mechanical ventilation	Grade IIIb
#42	Excessive overinfusion leading to abdominal compartment syndrome and facial edema requiring 20 hours postoperative mechanical ventilation	Grade IIIb
#50	Renal failure requiring renal replacement therapy (CVVHDF)	Grade IVa
#51	Massive facial edema/mucosal bleeding and respiratory distress requiring difficult airway management re-ETI and oral tamponade	Grade IVa
#52	Massive groin hematoma due to dislocation of arterial introducer requiring premature termination of PHMP	Grade IIIa
#53	Neck swelling prior procedure during CVC placement requiring postponing of intervention and extubation	Grade IIIa

ETI, endotracheal intubation; CVVHDF, continuous veno-venous hemodiafiltration; PHMP, percutaneous hepatic melphalan perfusion; CVC, central venous catheter

### Statistical analysis

Statistical analyses involved mainly descriptive analyses using numbers (percentages) or medians (ranges). Overall hemodynamic and laboratory courses were analyzed. The distribution of the continuous variables was tested by using the Shapiro–Wilk normality test. These variables are described as the mean and standard deviation (SD). The laboratory courses were analyzed by using analysis of variance (ANOVA), if the data were normally distributed; otherwise, the Friedman test was used. To detect habituation effects, ANOVAs were performed and included the patients who received at least two PHMPs. P-values are provided as 2-sided values and were considered to be statistically significant below 0.05. A multivariable analysis was not performed due to the small sample sizes.

All of the statistical analyses were performed by using R software version 3.6.1 (R Foundation for Statistical Computing, Vienna, Austria).

## Results

### Patients’ characteristics

Within the observation period, 16 patients (11 female and 5 male patients), with a median age of 60 years (range: 34–81 years), underwent 53 PHMP procedures. The baseline patient characteristics are described in Table 1. Patients underwent a median of two PHMP procedures (range: 1–6 procedures). Five patients received one procedure each, three patients received two procedures each, one patient received three procedures, three patients received five procedures each, and four patients received six procedures each. The procedure times can be found in Table 2. The times of the anesthesia procedures (induction and CVC placements) exceeded 60 minutes in five cases, due to mechanical complications of the CVC placements. In most cases, ICU discharge was possible after one day (n = 46; 87% of cases), whereas seven cases required two days of ICU stay and one case required three days of ICU stay.

### Anesthesia

Anesthesia induction was performed by using sufentanil (0.4–0.8 μg kg^-1^) in 28 cases and by using remifentanil (0.15–0.4 μg kg min^-1^) in 25 cases. In one case, sufentanil was used for induction and remifentanil was used for the maintenance of anesthesia. Propofol (1.0–2.0 mg kg^-1^) and rocuronium (0.6–1.0 mg kg^-1^) were used in all of the cases. After induction, patients underwent balanced anesthesia (sevoflurane n = 30, isoflurane n = 14) or total intravenous anesthesia (TIVA) by using propofol (n = 9).

### Airway management and mechanical ventilation

In all of the cases, first-pass success tracheal intubation was achieved following Cormack and Lehane (CL) grade I or II laryngoscopy conditions. Postoperative extubation in the fluoroscopic suite was possible in the majority of cases (n = 42; 79% of cases). In 11 cases, patients were admitted to the ICU under mechanical ventilation, due to prolonged vasopressor requirements, edema, or new incidences of anisocoria requiring further computed tomography (CT) diagnostic procedures. One patient underwent reintubation in the ICU due to progressive neck swelling (as described below). The median time of postoperative ventilation in the ICU was 4 hours (range: 1–20 hours). In the ICU, oxygen was necessary in 39 cases (74% of cases), with oxygen being supplied in 21 cases for a median of 7 hours (range: 3–14 hours) and in 18 cases until ICU discharge.

### Perioperative complications

In 33 PHMP procedures (62%), we observed a total of 84 complications and adverse events until ICU discharge. Most of these adverse events were minor, whereas nine major complications (grade III and IV) were observed (Table 3).

#### Airway

Perioperative hypoxic events, which were defined as SpO_2_ values below 90%, were observed in two cases (SpO_2_ 82% and 73%). The first patient developed hemodynamic instability, pulmonary edema, and bronchospasms during melphalan washout, which required high-dose noradrenaline and adrenaline administrations, as well as high-flow pure oxygen ventilation, fenoterol inhalation, and methylprednisolone administration. In the ICU, the patient developed acute renal failure that required renal replacement therapy (RRT) but was transferred to the normal ward after three days. The other patient had already been extubated after sufficient spontaneous breathing but required immediate reintubation in the fluoroscopic suite, due to desaturation caused by rapidly progressing facial edema, as well as oral and nasal bleeding. Bag-mask ventilation was possible by using an oropharyngeal tube. Two attempts of direct laryngoscopy presented with CL grade IV conditions, and tracheal intubation was performed by using videolaryngoscopy (VLS) and an introducing guide. After treatment with oral tamponade, topical adrenalin dressings, methylprednisolone administration, and furosemide administration, tracheal extubation was possible after 16 hours, and the patient was able to be discharged from the ICU after two days. Another patient required reintubation at the ICU, due to progressive neck swelling (as described below).

#### Vascular catheterization

Right IJV catheterization (two punctures for the CVC and the sheath) was performed in 49 cases, whereas right and left IJV catheterizations (one puncture each) were performed in four cases. Mechanical complications related to CVC placement were observed in 11 cases (21% of cases) (most of which were minor), including multiple puncture approaches due to intravascular thrombosis and/or an inability to advance the guidewire. Inadvertent carotid artery puncture was not observed. In nine cases, right-sided neck swelling was observed after the procedure (no left-sided swelling was noted). Two patients experienced critical CVC puncture-related complications. One patient required multiple puncture approaches to the right and left internal jugular veins, due to the inability to advance the guidewire. Finally, after successful CVC placement, the patient developed cervical hematoma, and PHMP was postponed for safety reasons. The patient was weaned off and extubated after the removal of the CVC devices in the fluoroscopic suite. Two weeks after the incident, the patient underwent PHMP without any complications. The other patient experienced apparently normal CVC placements without visible neck swelling during the procedure. Six hours after extubation and admittance to the ICU, progressive neck swelling and dyspnea required emergent bedside reintubation by using VLS. Computed tomography revealed two small active bleeding events of the thyrocervical trunc, which were treated by angiography by using histoacryl occlusion. The patient was able to be extubated on the same day and was discharged to the regular ward after two days. The same patient underwent another five PHMPs without complications related to vascular access.

Complications regarding arterial catheterizations were observed in one patient who developed massive hematomas of the inguinal region, right upper thigh, and scrotum after the dislocation of the arterial introducer in the common femoral artery. The PHMP procedure was prematurely terminated, and the patient recovered without further treatment.

#### Hemodynamics

**Fig 1:**
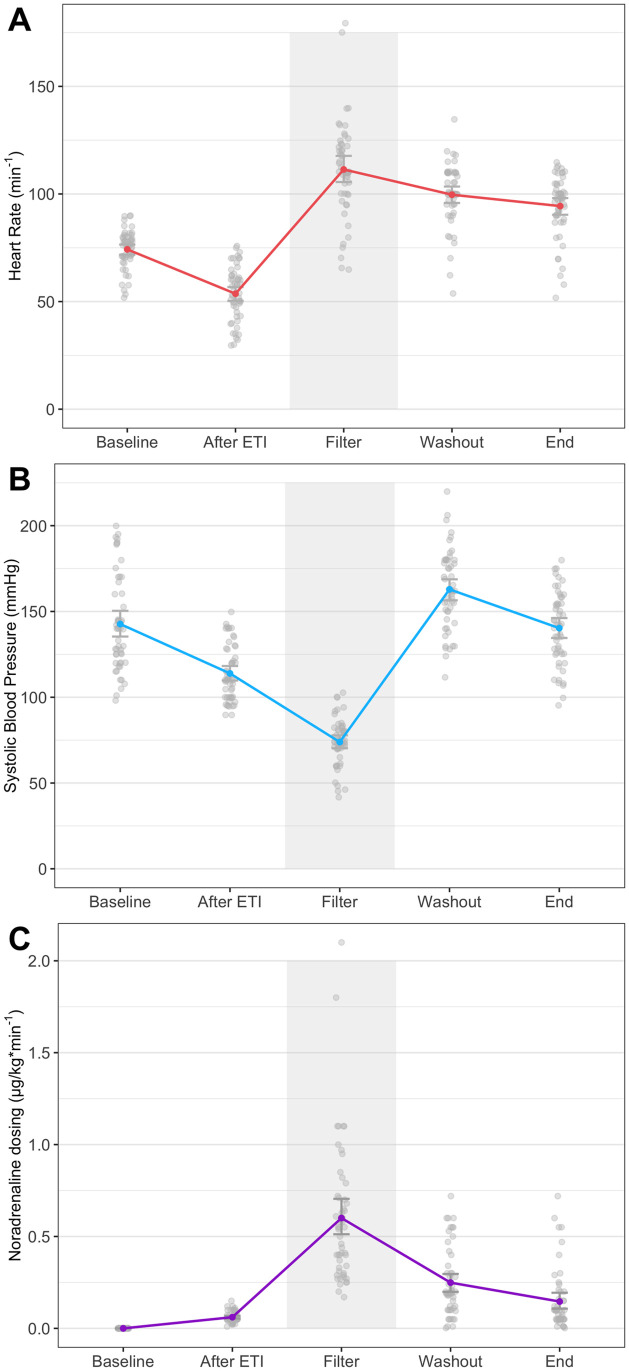
Hemodynamics and noradrenaline dosage at procedure steps.

**Fig 2:**
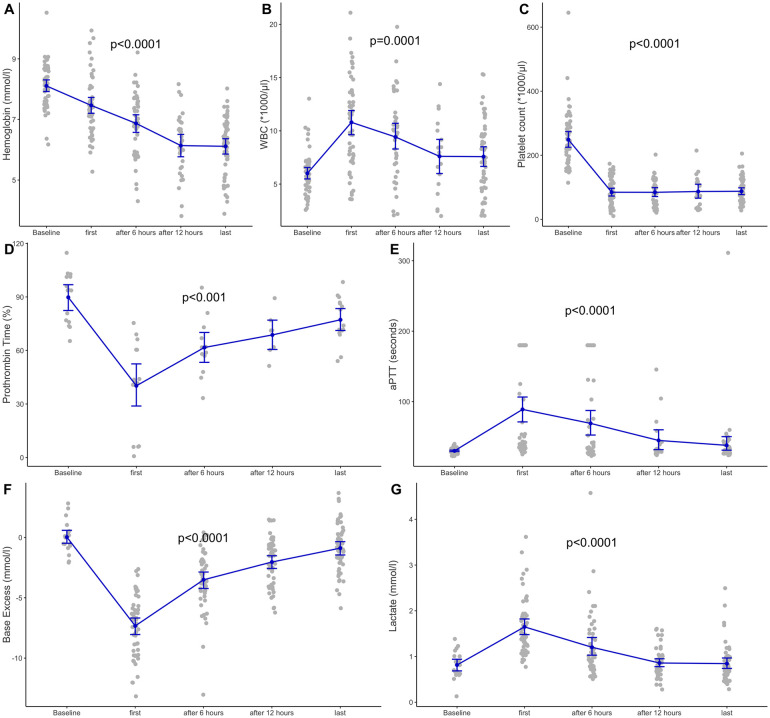
Laboratory course until intensive care unit discharge.

The hemodynamic course during PHMP was associated with considerable variability in either SBP or heart rate (Fig 1). In seven cases, patients experienced transient severe arterial hypotension (SBP <60 mmHg), and eight cases showed transient arterial hypertension (SBP >200 mmHg), mainly due to noradrenaline rebound effects. In nine cases, patients experienced bradycardia (HR <40/min^-1^); in three cases, patients had tachycardia (HR >140/min^-1^). During anesthesia induction, bolus administrations of theodrenaline/cafedrine (Akrinor^™^) were performed in 19 cases (36% of cases). After CVC placement, all of the patients received continuous noradrenaline infusion. Until melphalan administration, only low noradrenaline doses (<0.01 μg kg min^-1^) and low fluid volumes were required. After the blockade of the inferior cava vein via insufflation of both inserted balloons, hypotension occurred in 45 cases (85% of cases). During the start of extracorporeal circulation and the wash-in of melphalan, all of the patients became increasingly hypotensive within a few minutes and developed tachycardia in 50 cases (94% of cases). The highest median rate of noradrenaline was 0.5 μg kg min^-1^ (range: 0.17–2.1 μg kg min^-1^), whereas dosages of >0.5 μg kg min^-1^ were observed in 23 cases (43% of cases), including seven cases requiring >1 μg kg min^-1^. Repeated bolus administrations of noradrenaline were required in 38 cases (72% of cases), whereas additional bolus administrations of adrenaline were required in two cases. The median crystalloid infusion volume during PHMP was 5 L (range: 3–14 L); in 13 cases (24% of cases), patients received ≥8 L, including 4 cases exceeding 10 L. In two cases, patients received albumin during the intervention. In the ICU, noradrenaline was required in 15 cases (28% of cases), of which nine cases were mechanically ventilated; additionally, larger volumes of fluid administration were not required. Perioperative overinfusion led to transient intestinal edema and abdominal distention in one case, and considerable facial edema was observed in four cases, including the case in which emergent reintubation was required. Arterial blood gas analyses revealed significant decreases in base deficit and lactate levels from ICU admission until discharge (Fig 2).

#### Coagulation management, transfusion requirement, and bleeding complications

In two-thirds of cases (n = 36; 68% of cases), heparin was reversed by using protamin (median: 25,000 units). Perioperatively, no transfusion was performed, whereas AT III was administered in two cases, and fibrinogen was administered in one case. In the ICU, patients received blood products in six cases (Table 4). Bleeding complications that were not associated with vascular catheterization were macrohematuria (two cases), epistaxis, and oropharyngeal bleeding (six cases). Coagulation markers significantly recovered during the ICU stay, whereas hemoglobin showed considerable hemodilution effects (Fig 2).

**Table 4:** Postoperative transfusion requirements until intensive care unit discharge.

Procedure	Transfusion specifics
#11	One platelet concentrate
#22	Three FFP
#37	One platelet concentrate and one FFP
#50	Three FFP and 4g fibrinogen
#51	Three FFP
#52	One RBC
#53	Two platelet concentrates

FFP, fresh frozen plasma; RBC, red blood cell concentrate

#### Other complications

Preoperative and postoperative urinary output levels were normal in all but four patients. One patient developed acute renal failure requiring RRT (as mentioned above), another patient had acute urinary retention after bladder catheter removal that required prolonged urinary catheterization, and two patients had macrohematuria (as mentioned above), which disappeared until ICU discharge.

We observed one balloon rupture and two balloon displacements during PHMP, all of which were able to be managed without further sequelae. One patient developed new anisocoria after PHMP and required a head CT and prolonged mechanical ventilation to rule out intracranial bleeding. The patient recovered well without any deficits and the reason for anisocoria remained unclear. In five cases, peripheral neurological impairment and dysesthesia were observed, including transient palsy of the right hand due to improper perioperative positioning (n = 1 case), upper thigh dysesthesia due to puncture-related thigh hematoma (two cases), and progressive face edema (two cases). All of the patients recovered well during their stays in the ICU.

In nine cases (17% of cases), nausea occurred within their first hours after ICU admission, including four cases that exhibited significant vomiting. All of the patients received either ondansetron or dimenhydrinate.

**Fig 3:**
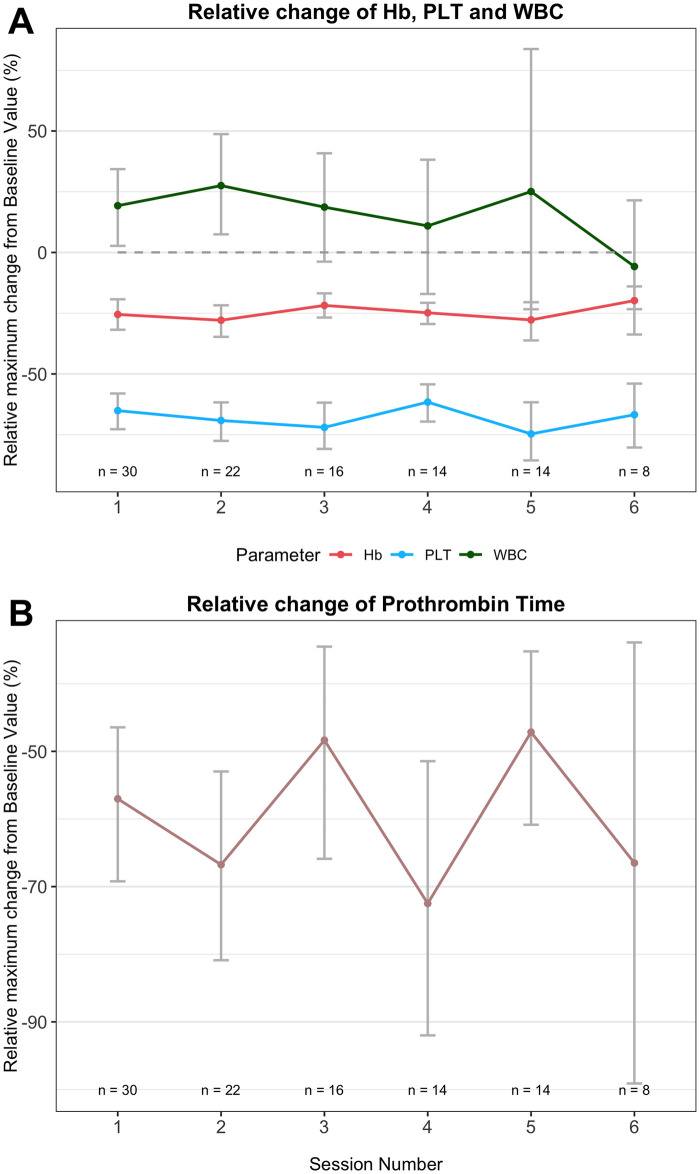
Habituation effect analysis.

Three patients complained of diffuse cervical and abdominal pressure, as well as palpitations (which were regredient in all cases). One patient developed angina pectoris symptoms, and acute coronary syndrome was ruled out via repeated electrocardiography and troponin tests. One case of anaphylactic reaction to allopurinol was successfully treated with fluid resuscitation and hydrocortisone. In four cases, patients developed perioperative hypothermia (<35°C), whereas febrile episodes (>38°C) were observed during the ICU stay in 20 cases (38% of cases), including three cases exceeding 39°C. WBC counts increased from baseline to PHMP and decreased during the ICU course, including four cases presenting with slight leukopenia at the time of ICU discharge (Fig 2). In patients undergoing more than one PHMC, no significant habituation effects of laboratory parameters were observed (Fig 3).

#### Response and outcomes

Response to PHMP with decrease of tumor size was observed in 13 patients (81%) including three patients with a tumor size decrease of >50%. Overall survival rate was 50%, whereas all fatalities were not associated with PHMP. Detailed oncological courses, interventional radiologists performances and long-term outcomes of the patients were subject of separate analyses of the study group.

## Discussion

Our results suggested that the perioperative management of PHMP is frequently associated with procedure-related issues. These findings are in similarity with previous studies from other centers [1–15]. The main issues related to PHMP were hemodynamic depression, vascular access complications, and coagulopathy. In two cases, mechanical complications led to the premature termination or postponement of PHMP. Although no fatalities occurred and almost all of the cases recovered well within one day, we observed potentially life-threatening adverse conditions. Difficult airway management, prolonged mechanical ventilation, cervical bleeding requiring endovascular intervention, and renal failure requiring renal replacement therapy were the most challenging conditions. Based on our data, we are not able to conclude whether these complications are unavoidable.

The published prevalence of perioperative complications related to PHMP is not easy to assess, due to multiple reporting [4–12] and multicenter approaches [10, 12]. Although no direct fatalities during PHMP have been reported in the literature, one patient suffered cardiac arrest due to coronary disease with successful cardiopulmonary resuscitation [13]. Cardiac arrhythmia related to PHMP includes asystoly, ventricular fibrillation, ventricular tachycardia, supraventricular tachycardia, and total atrioventricular block [1, 11, 12, 15]. One patient has been observed to die of a retroperitoneal giant hematoma 30 hours after PHMP, which was likely related to heparin-induced coagulopathy [11]. Two patients have been shown to die at 3 days and at 12 days after PHMP, due to liver failure [8]. Another patient developed severe multiorgan failure during PHMP and died 48 days post-operation [10].

Due to our experiences, anesthesia appeared to be safe to use via either balanced anesthesia or total intravenous anesthesia (TIVA), and we did not observe the need for higher dosages of propofol, due to possible filtration during PHMP. PHMP-associated complications led to reintubation in two cases, which was associated with impaired views in direct laryngoscopy due to cervical edema and/or bleeding. Thus, difficult airway management should always be anticipated.

Hemodynamic instability, particularly during balloon occlusion and melphalan wash-in, required advanced anesthetic expertise. We frequently observed large intraindividual ranges of SBP and heart rate, which were similar to previous studies that have reported significant transient hypotension during hemofiltration [1–15]. Hence, patients scheduled for PHMP should present with appropriate cardiovascular fitness and without pathologies of the brain-supplying vessels to avoid cerebral ischemia.

The circulatory response to the artificial surfaces of the extracorporeal circulation unit may be encountered by appropriate preoperative intravascular filling and increases in the noradrenaline dosages prior to the start of the pump [2]. Furthermore, the toxicity of melphalan may be less pronounced when improved filter systems are used, which are more effective in avoiding unintended systemic melphalan stream-in events than first-generation filters [3].

In the literature, detailed data concerning circulatory management during PHMP are scarce. Most studies have mentioned rather general treatment strategies (vasopressor use and fluid resuscitation) [1, 3–15]. In one study, PHMP patients received infusion volumes of up to eight liters, but no further details were provided [13]. Despite our aim to primarily use noradrenaline for hypotension control, some cases required massive additional fluid resuscitation volumes. However, overhydration carries the risk of secondary complications (edema, ascites, pleural effusion, respiratory compromises, and coagulation disorders due to hemodilution) [8, 12]. Studies involving advanced hemodynamic monitoring (i.e., thermodilution systems) to guide individual fluid volume administration and vasopressor dosage during PHMP are not available but may contribute to the safety of this procedure.

The coagulation management of PHMP may further aggravate cardiovascular complications. Although high-dose heparin administration is mandatory for safe extracorporeal circulation and bleeding complications may arise if it is not reversed, protamin administration carries the risk of thromboembolic complications. As a consequence of experiencing thromboembolic events, some centers now avoid protamin administration, in contrast to recommendations [2, 8]. In our center, we have changed our strategy from the rigid use of protamin administration to thorough case-by-case decisions.

Unlike other studies, we did not observe major thromboembolic events (myocardial infarction or cerebrovascular stroke) in our patients. Published thromboembolic complications include strokes ranging from complete resolution without any neurological deficits [8, 10] to persistent symptoms despite uses of immediate thrombectomy [8], as well as incidences of pulmonary embolism [5, 15] and cardiac ischemia [1, 5, 14, 15].

In our patient cohort, all of the neck swelling complications were related to CVC placement, with one case requiring endovascular intervention. The unintentional puncture of the carotid artery, which may have resulted in potentially life-threatening hematoma formation after heparin administration, was prevented by the use of direct ultrasound visualization in all cases.

In the literature, bleeding due to vascular catheter-related complications or heparin administration has been observed in up to 30% of cases [5, 15]. Bleeding events included groin hematoma [10], puncture site hematoma [10, 12], retroperitoneal hematoma [11], transfemoral bleeding (requiringing surgery) [14], femoral pseudoaneurysma (requiring surgery) [8, 10], aneurysma spurium [12], the dissection of the common hepatic artery (requiring prolonged angioplasty) [8], gastric ulcerous bleeding in two patients (one requiring surgery) [8], intraocular hemorrhage [8], epistaxis [5, 8], vaginal bleeding [5, 11], and simultaneous abdominal bleeding and cerebral hemorrhage [15].

We observed one case of renal failure requiring RRT and prolonged ICU LOS. In the literature, acute renal failure after PHMP has been reported without RRT [10]. However, data on the possible RRT of at least four patients who died from organ failure in close proximity to PHMP were not available [8, 10, 11]. There remains a risk of underestimations and an underreporting of PHMP-associated complications, due to study designs, incomplete assessments, divergent definitions, and possible conflicts of interest.

Uveal melanoma, the main underlying disease of the patients in this study, is known for aggressive progression of unresectable liver metastases. Recent studies reveal that survival rates are still poor [18, 19]. In contrast to cutaneous melanoma treatment, the promising effects of immune checkpoint inhibitors may be not applicable to uveal melanoma treatment and further research is needed [20].

Our study confirms that PHMP may be a promising non-surgical treatment option to decrease the volume of liver tumors and metastases in selected patients. Respondents may benefit from PHMP, whereas expert perioperative management and thorough postoperative care are key to maintain successful results.

### Limitations

We acknowledge the weaknesses of retrospective cohort studies, including long observational periods and low sample sizes. Thus, the number of complications may have been higher due to documentation issues. In this study, we presented data from a single center, which may not be comparable to other centers, due to different settings and treatment modalities. Previous reports of the anesthetic management of PHMP procedures are not available; thus, our data are descriptive and hypothesis-generating, rather than conclusive.

### Conclusions

Percutaneous hepatic melphalan perfusion procedures are frequently associated with challenging circulation conditions and serious mechanical complications that require advanced peri- and postoperative managements. For safety reasons, it should only be performed in selected patients by experienced operators in specialized hospital infrastructures. Future studies should evaluate the feasibility of perioperative advanced hemodynamic monitoring for the estimation of optimal individual fluid resuscitation and vasopressor ratios.

## Supporting information

S1 Data Raw dataset. (XLSX)

## References

[1] Hughes MS, Zager J, Faries M, Alexander HR, Royal RE, Wood B, et al. Results of a Randomized Controlled Multicenter Phase III Trial of Percutaneous Hepatic Perfusion Compared with Best Available Care for Patients with Melanoma Liver Metastases. Ann Surg Oncol. 2016;23: 1309–1319. doi: 10.1245/s10434-015-4968-3 26597368 PMC8185532

[2] CHEMOSAT hepatic delivery system for melphalan hydrochloride for injection. Instructions for use. 2014. Delcath systems inc. 566 Queensbury Avenue Queensbury, NY 12804 USA

[3] Forster MR, Rashid OM, Perez MC, Choi J, Chaudhry T, Zager JS. Chemosaturation with percutaneous hepatic perfusion for unresectable metastatic melanoma or sarcoma to the liver: a single institution experience. J Surg Oncol. 2014;109: 434–439. doi: 10.1002/jso.23501 24249545 PMC4503311

[4] Meijer TS, Burgmans MC, de Leede EM, de Geus-Oei LF, Boekestijn B, Handgraaf HJM, et al. Percutaneous Hepatic Perfusion with Melphalan in Patients with Unresectable Ocular Melanoma Metastases Confined to the Liver: A Prospective Phase II Study. Ann Surg Oncol. 2021;28: 1130–1141. doi: 10.1245/s10434-020-08741-x 32761328 PMC7801354

[5] Meijer TS, Burgmans MC, Fiocco M, de Geus-Oei LF, Kapiteijn E, de Leede EM, et al. Safety of Percutaneous Hepatic Perfusion with Melphalan in Patients with Unresectable Liver Metastases from Ocular Melanoma Using the Delcath Systems’ Second-Generation Hemofiltration System: A Prospective Non-randomized Phase II Trial. Cardiovasc Intervent Radiol. 2019;42: 841–852. doi: 10.1007/s00270-019-02177-x 30767147 PMC6502784

[6] de Leede EM, Burgmans MC, Meijer TS, Martini CH, Tijl FGJ, Vuyk J, et al. Prospective Clinical and Pharmacological Evaluation of the Delcath System’s Second-Generation (GEN2) Hemofiltration System in Patients Undergoing Percutaneous Hepatic Perfusion with Melphalan. Cardiovasc Intervent Radiol. 2017;40: 1196–1205. doi: 10.1007/s00270-017-1630-4 28451811 PMC5554291

[7] Dewald CLA, Hinrichs JB, Becker LS, Maschke S, Meine TC, Saborowski A, et al. Chemosaturation with Percutaneous Hepatic Perfusion: Outcome and Safety in Patients with Metastasized Uveal Melanoma. Rofo. 2021 Feb 3. doi: 10.1055/a-1348-1932 33535258

[8] Schönfeld L, Hinrichs JB, Marquardt S, Voigtländer T, Dewald C, Koppert W, et al. Chemosaturation with percutaneous hepatic perfusion is effective in patients with ocular melanoma and cholangiocarcinoma. J Cancer Res Clin Oncol. 2020;146: 3003–3012. doi: 10.1007/s00432-020-03289-5 32564137 PMC7519914

[9] Kirstein MM, Marquardt S, Jedicke N, Marhenke S, Koppert W, Manns MP, et al. Safety and efficacy of chemosaturation in patients with primary and secondary liver tumors. J Cancer Res Clin Oncol. 2017;143: 2113–2121. doi: 10.1007/s00432-017-2461-z 28634727 PMC11819086

[10] Marquardt S, Kirstein MM, Brüning R, Zeile M, Ferrucci PF, Prevoo W, et al. Percutaneous hepatic perfusion (chemosaturation) with melphalan in patients with intrahepatic cholangiocarcinoma: European multicentre study on safety, short-term effects and survival. Eur Radiol. 2019;29: 1882–1892. doi: 10.1007/s00330-018-5729-z 30255257

[11] Vogl TJ, Zangos S, Scholtz JE, Schmitt F, Paetzold S, Trojan J, et al. Chemosaturation with percutaneous hepatic perfusions of melphalan for hepatic metastases: experience from two European centers. Rofo. 2014;186: 937–44. doi: 10.1055/s-0034-1366081 24729409

[12] Vogl TJ, Koch SA, Lotz G, Gebauer B, Willinek W, Engelke C, et al. Percutaneous Isolated Hepatic Perfusion as a Treatment for Isolated Hepatic Metastases of Uveal Melanoma: Patient Outcome and Safety in a Multi-centre Study. Cardiovasc Intervent Radiol. 2017;40: 864–872. doi: 10.1007/s00270-017-1588-2 28144756

[13] Artzner C, Mossakowski O, Hefferman G, Grosse U, Hoffmann R, Forschner A, et al. Chemosaturation with percutaneous hepatic perfusion of melphalan for liver-dominant metastatic uveal melanoma: a single center experience. Cancer Imaging. 2019 May 30;19:31. doi: 10.1186/s40644-019-0218-4 31146793 PMC6543599

[14] Brüning R, Tiede M, Schneider M, Wohlmuth P, Weilert H, Oldhafer K, et al. Unresectable Hepatic Metastasis of Uveal Melanoma: Hepatic Chemosaturation with High-Dose Melphalan-Long-Term Overall Survival Negatively Correlates with Tumor Burden. Radiol Res Pract. 2020 Sep 2;2020:5672048. doi: 10.1155/2020/5672048 32934846 PMC7484678

[15] Karydis I, Gangi A, Wheater MJ, Choi J, Wilson I, Thomas K, et al. Percutaneous hepatic perfusion with melphalan in uveal melanoma: A safe and effective treatment modality in an orphan disease. J Surg Oncol. 2018;117: 1170–1178. doi: 10.1002/jso.24956 29284076 PMC6033148

[16] Woodman SE. Metastatic uveal melanoma: biology and emerging treatments. Cancer journal (Sudbury, Mass). 2012;18: 148–52. doi: 10.1097/PPO.0b013e31824bd256 22453016 PMC3935729

[17] McEwan PE, Bailey L, Trost D, Scull C, Keating JH, Williams M, et al. Percutaneous Hepatic Perfusion With Filtered Melphalan for Localized Treatment of Metastatic Hepatic Disease: A Risk Assessment. Int J Toxicol. 2018;37: 434–447. doi: 10.1177/1091581818811306 30453808

[18] Lane AM, Kim IK, Gragoudas ES. Survival Rates in Patients After Treatment for Metastasis From Uveal Melanoma. JAMA Ophthalmol. 2018;136: 981–986. doi: 10.1001/jamaophthalmol.2018.2466 29955797 PMC6142974

[19] Johnson DB, Daniels AB. Continued Poor Survival in Metastatic Uveal Melanoma: Implications for Molecular Prognostication, Surveillance Imaging, Adjuvant Therapy, and Clinical Trials. JAMA Ophthalmol. 2018;136: 986–988. doi: 10.1001/jamaophthalmol.2018.1813 29955760

[20] Wessely A, Steeb T, Erdmann M, Heinzerling L, Vera J, Schlaak M, et al. The Role of Immune Checkpoint Blockade in Uveal Melanoma. Int J Mol Sci. 2020;21:879. doi: 10.3390/ijms21030879 32013269 PMC7037664

