# Correction: Percutaneous hepatic melphalan perfusion: Single center experience of procedural characteristics, hemodynamic response, complications, and postoperative recovery

**DOI:** 10.1371/journal.pone.0314055

**Published:** 2024-11-13

**Authors:** Manuel Florian Struck, Peter Kliem, Sebastian Ebel, Alice Bauer, Holger Gössmann, Rhea Veelken, Florian van Bömmel, Timm Denecke, Sebastian N. Stehr, Felix F. Girrbach

The eighth author’s name is spelled incorrectly. The correct name is: Timm Denecke.
